# Detection and Identification of Multiple Stationary Human Targets Via Bio-Radar Based on the Cross-Correlation Method

**DOI:** 10.3390/s16111793

**Published:** 2016-10-27

**Authors:** Yang Zhang, Fuming Chen, Huijun Xue, Zhao Li, Qiang An, Jianqi Wang, Yang Zhang

**Affiliations:** 1 Department of Biomedical Engineering, Fourth Military Medical University, Xi’an 710032, China; zyfmmu@126.com (Ya.Z.); cfm5762@126.com (F.C.); xinyin20130419@163.com (H.X.); lizhaofmmu@fmmu.edu.cn (Z.L.); anqiang900903@163.com (Q.A.); 2 Center for Disease Control and Prevention of Guangzhou Military Region, Guangzhou 510507, China

**Keywords:** ultra-wideband radar, false positive and negative identification, multiple stationary human targets, energy detection, cross-correlation

## Abstract

Ultra-wideband (UWB) radar has been widely used for detecting human physiological signals (respiration, movement, etc.) in the fields of rescue, security, and medicine owing to its high penetrability and range resolution. In these applications, especially in rescue after disaster (earthquake, collapse, mine accident, etc.), the presence, number, and location of the trapped victims to be detected and rescued are the key issues of concern. Ample research has been done on the first issue, whereas the identification and localization of multi-targets remains a challenge. False positive and negative identification results are two common problems associated with the detection of multiple stationary human targets. This is mainly because the energy of the signal reflected from the target close to the receiving antenna is considerably stronger than those of the targets at further range, often leading to missing or false recognition if the identification method is based on the energy of the respiratory signal. Therefore, a novel method based on cross-correlation is proposed in this paper that is based on the relativity and periodicity of the signals, rather than on the energy. The validity of this method is confirmed through experiments using different scenarios; the results indicate a discernible improvement in the detection precision and identification of the multiple stationary targets.

## 1. Introduction

Ultra-wideband (UWB) radar is a special type of radar that is widely used for the detection and localization of people in critical situations and environments [1,2]. Owing to its good penetration ability and high spatial resolution, it can detect vital signs like breath, heartbeat, and movement, contactless through common materials (e.g., cloth, walls, rubble, and non-metallic obstacles) [3,4,5,6]. Hence, UWB radar is used for various applications such as detecting living persons trapped beneath the ruins after an earthquake, identifying the indoor distribution of people before hostage rescue in counter terrorism, and monitoring critical patients’ breath and heartbeat particularly, those for whom wiring electrodes, sensors, etc., are unsuitable [7,8,9]. The UWB radar which is mainly used for detecting breath, heartbeat and other biological signals is generally referred to as bio-radar. Owing to its prospective and desirable advantages, the bio-radar has been the focus of several research groups who have proposed new algorithms and prototypes for detecting respiration and for locating the position of the target precisely [10,11,12].

In the field of human detection via bio-radar, the number and location of the targets are the critical issues of concern that are yet to be fully resolved. Some research aims at tracking moving human targets behind the wall by extracting the doppler feature [13,14]. Owing to micro-Doppler effect, changes in the properties of the echo signal reflected from the moving target is more than that reflected from stationary one. The only body variations of stationary target are caused by the respiratory motion and heartbeat. Because the heart motion is so weaker than respiratory motion that the detection of heart motion is of less importance. The expansion and contraction of the chest cavity creates a visible change in the range profile because the reflected fraction of the energy of the electromagnetic waves incident on dry skin is approximately 72%, denoting the coefficient of reflectivity of the air-to-dry skin interface for electromagnetic waves in the 300–900 MHz range [10]. Hence, respiratory motion is used for detecting human targets [15]. Conventional methods of life detection are based on the energy detection of the respiration response [16,17,18,19]. However, these methods include certain problems that need to be resolved. It is difficult to identify human targets by the energy of the respiration response, when the signal-to-noise-and-clutter ratio (SNCR) is low as a result of the low reflectivity of the human body and the strong backscattered responses from the obstacles in the rubble [16]. Next, when multiple stationary human targets exist in the detection area, the respiration energy of the closer target is considerably stronger than that of the farther target, rendering the detection of the farther target difficult. This is mainly attributed to two factors. First, the energy of electromagnetic waves decreases with the increasing of the distance; hence, the energy of the electromagnetic waves reaching the farther target is inevitably subtler than those reaching the closer target [20]. Further, a shadow effect occurs when a farther target is located in the shadow, defined as the absence of radar illumination owing to presence of a closer target within the illuminating radar beam [21]. The shadow behind the target is caused by the reflection or absorption of the emitted radar waves by the target. It creates an area, wherein, there is a high attenuation of the electromagnetic wave energy behind a human target [22], rendering the other targets located in this area undetectable. In conclusion, the traditional life detection method based on the energy of the respiratory response is only suitable for a high SNCR, and the problems with the false negatives and positives are particularly significant when detecting multiple stationary human targets. To solve this problem, a novel target detection and identification method that does not rely on the energy of the respiratory response has been examined.

As previously known, the respiration signals in a radar echo have the following characteristics [23]: (i) The geometrical changes in the chest during breathing are considerably smaller than the range resolution of a convention radar; therefore, the response from the breathing target is displayed at different phases of the respiratory motion. (ii) The distance between the radar and the target does not change during detection regardless of body movement, i.e., the breathing always appears at constant moment in the propagation time. (iii) Breathing appears in several neighboring cells for the reasons that physical size of human body is not ignorable and the tailing effect exists. In conclusion, if breathing appears at a certain moment in the propagation time, an area with a high correlation turns up where the breathing appears. This correlation is closely related to the periodicity of breathing instead of its energy. Even if noise or clutter exists at the same frequency as that of the breathing, they are more likely to be random and independent, and correlation areas do not appear after them. Cross-correlation is a standard method of estimating the degree of correlation between two series. Some studies have reported the application of cross-correlation for human detection [24,25]. In [24], two identical microwave systems are applied to receive echoes simultaneously and the results after a cross-correlation analysis between the signals from the two receivers show that the noises are suppressed, whereas, the energies of the vital signs of life are accumulated because the signals are correlated and the noises are uncorrelated. The signal-to-noise ratio is improved. In [25], based on the assumption that the respiration of a target detected from different channels are highly correlated, and the correlation between the respiration of different targets and the noises are low among the channels, a correlation analysis is used to restrain the noises and distinguish the human targets. All the above correlation processing has been done between the radar echoes from different channels; however, correlation analysis between signals from a single channel have not yet been reported. A novel target detection and identification method is proposed in this paper, mainly based on the property that there is a high correlation area behind each target, and the count and location of the targets can be confirmed by recognizing the correlation area. This method is applied for target detection and identification for the first time. The problems of the false negatives and positives in a multiple stationary human target detection scenario are resolved effectively. The performance of this new method will be proved by experiments using different scenarios.

To solve the above described problem, a bistatic Impulse Radio(IR) UWB bio-radar system is developed in Section 2. A pre-processing algorithm is implemented for improving the signal-to-noise (SNR) of the echo from radar, as described in Section 3. After pre-processing, a method based on cross-correlation is proposed for accurately identifying and locating different targets in Section 4. Finally, the experimental results for different scenarios are presented and discussed in Section 5, and the paper is concluded in Section 6.

## 2. Bio-Radar System

**Figure 1:**
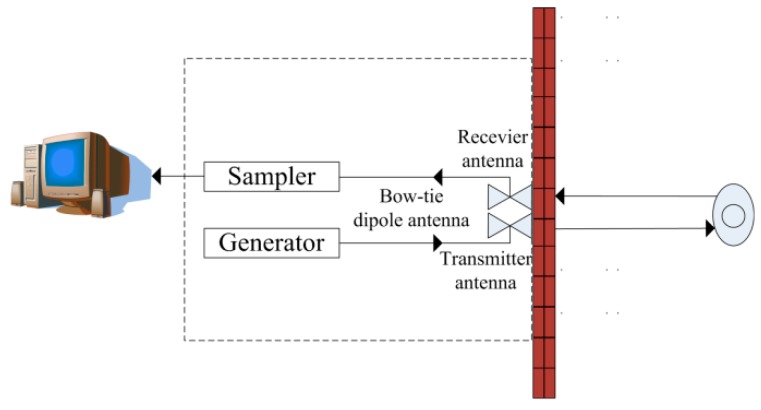
Bistatic IR-UWB bio-radar setup.

The bio-radar setup is shown in Figure 1. Bow-tie dipole antennas, commonly referred to as the transmitter antenna and the receiver antenna, stuck on the wall using Velcro are employed for the transmission and reception of the electromagnetic waves. Pulses generated by a pulse generator with a pulse-repetition frequency (PRF) of 128 kHz are sent to the transmitter antenna for exciting the bow-tie dipole antenna. The echo pulses are received by the receiver antenna with a center frequency and bandwidth of 500 MHz each. Further, the echo pulses are input to a sampler and sampled at selective ranges according to range gate generated by a delay circuit and a range gate generator. Note that the transmitted pulses and the delay are produced by the same oscillator.

The echo signals are then stored in the form of waveforms after amplification and integration by a signal processor. A data matrix $H_{M\;\times \;N}$ is generated, where M denotes the sampling point in propagation time and N denotes the sampling point in observation time. The waveforms contain M=2048 sample points and the recorded profile is $\tau_{\max}=60$ ns long. The time-axis along each received waveform is termed as the “fast-time” and denoted by τ that is in the order of nanoseconds and on behave of range information. In practice, this time window is adjustable according to detection range of the radar. The time interval between each successive received waveform is $T_{s}=7.8\times 10^{-6}$ s. The time-axis along the interval is termed as the “slow-time” and is denoted by t that is in the order of seconds and on behave of time information. The commonly used monitoring time is T=60 s and the sampling frequency in the slow-time is $F_{s}=64\;\mathrm{Hz}$, which is greater than the Nyquist sampling rate for the respiration and heart signals, so the number of recorded waveform in slow-time is $N=T\times F_{s}=3840$. With the 500 MHz center frequency and 500 MHz band width, the range resolution of the system depends on waveform sample points, recorded profile, and signal-to-noise-and-clutter ratio (SNCR). When the waveform sample point *M* is selected as 2048, and the recorded profile *τ*_max_ is set as 60 ns (9 m in detect range), the theoretical range resolution will be up to 9/2048 = 4.4 mm. Consider the noise and clutter, generally, the range resolution can achieve centimeter magnitude which precise enough to detect the subtle motion of the human chest caused by respiration.Accordingly, the sampling frequency in the slow-time is $F_{s}=\frac{1}{T_{s}}=64$ Hz which is greater than the Nyquist sampling rate for the respiration and heart signals. These values are stored in a matrix. The received waveforms are measured at discrete time during the slow time, and the discrete-time sequences are sampled during every sampling period in the fast time.

## 3. Signal Pre-Processing and Analysis

The targets detected in our experiments are generally stationary and are located behind an obstruction such as a wall, and respiration is the primary information sensed by the bio-radar. Therefore, the algorithms described below mainly focus on the detection of the respiration. They can be divided into six steps: (i) The raw data, $H_{M\;\times \;N}$, is large in volume and time-consuming for a real-time operation; hence, it is sampled and accumulated along the fast-time dimension to shorten the length of the data. Generally, the raw data $H_{M\;\times \;N}$ are compressed into $H_{200\times N}$. (ii) The respiration frequency information is always accompanied by background clutter, comprising a multipath corresponding to the stationary scatterers in the surrounding; the method that has been employed to eliminate the background clutter involves the subtraction of the average of all the waveforms from each waveform, working as a “motion-filter” that separates the motional scatterers from the stationary scatterers. For the matrix $H_{200\times N}$, the mathematical expression of motion-filter is $T(m,n)=H(m,n)-\frac{1}{N}\sum_{i=1}^{N}H(m,i)$. (iii) As the respiration signal is a low frequency narrowband signal, a finite impulse response (FIR) filter is adopted along the slow-time dimension to suppress the high frequency noise. (iv) The energy of each waveform averaged over t is computed and the results show that the energy at the target location is larger than those at the other locations. Owing to the trailing effect, the energies of the areas behind the target are also large to a certain extent; this will pose a problem if the targets are located close to each other and a method of adaptive cancellation is applied to attenuate the trailing interferences between the targets [26]. After the above processing, a new matrix, $P_{200\times N}$, is obtained that can also be expressed as follows: $P=(p_{1},p_{2}\cdots p_{200})$, where $p_{i}$ denotes the row vector along the slow-time dimension and i is the index in the fast-time dimension. (v) After accumulating the filtered waveforms along the slow-time, the two-dimensional data including the range and time information is compressed into a one-dimensional range profile and the energy estimation of the bio-radar data has been obtained, indexing the fast-time bin indices. (vi) As the magnitude of the energy of the respiration components in the bio-radar echo signal obtained using the previous steps is significant, the target range needs to be located relying on distinguishing these maximum values in the energy estimation. As described earlier, a farther target could not be detected in a multiple stationary human target detection scenario owing to the shadowing effect.

**Figure 2:**
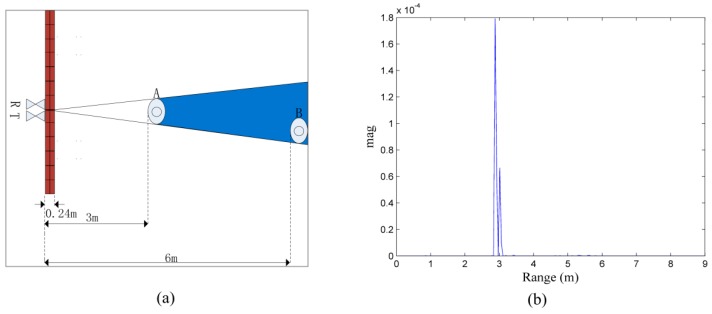
(**a**) scenario with two targets A and B; and (**b**) energy of the data received from the scenario.

As shown in Figure 2a, the farther target, B, is located in the shadowing area caused by the closer target, A. In Figure 2b, as the energy of the reflected signal from B is considerably weaker than the energy of the reflected signal from A, target B cannot be detected based on the energy detection of the respiratory response. To solve this problem, a new target identification method is proposed in this paper that does not rely on the energy of the reflected signal.

## 4. Cross-Correlation Analysis

**Figure 3:**
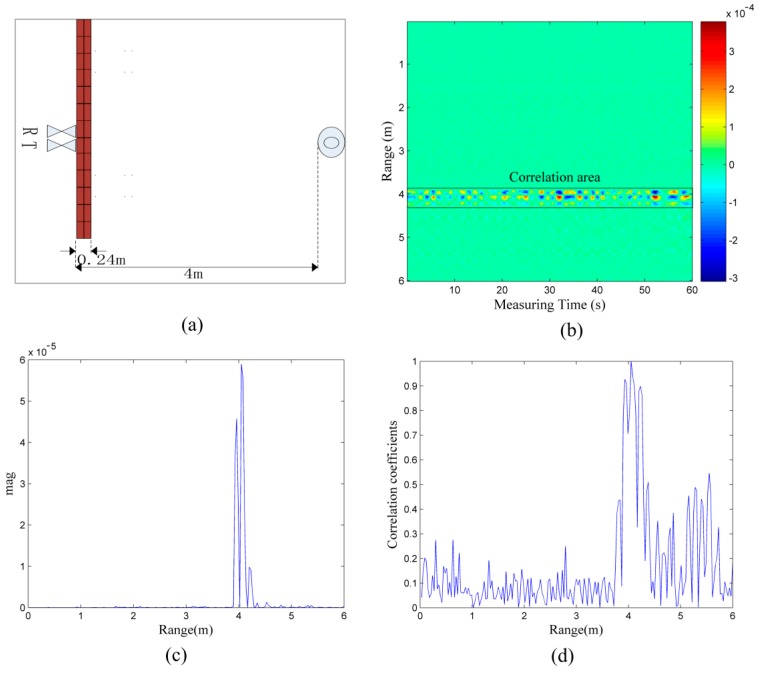
(**a**) scenario with a single target; (**b**) data received from the scenario with a single target as depicted in (**a**); (**c**) energy of the data in (**b**); and (**d**) correlation coefficients between the waveform at the peak energy location and the waveforms in the other locations.

As mentioned previously, breathing appears in several neighboring cells that have a high correlation with each other in the radar response. The width of the correlation area depends on the length of the impulse response of the antennas, the delay spread of the propagation path (rubble), physical size of the body which is moved during the respiration activity, position of body and ruble type, thickness and structure. For the single target in Figure 3a, there is a very obvious correlation area in the target location in Figure 3b. In Figure 3c, it can be seen that the signal has a peak energy exactly at the target location. The correlation coefficients between the signal with the peak energy and the remaining signals is shown in Figure 3d; the correlations between the signal with the peak energy and the signals in the correlation area are considerably larger than those of the others. In addition, the correlation between the target signal and the signals behind the target is high.

$p_{i}$ and $p_{j}$ denote the two received waveforms along the slow-time dimension, respectively. i and j are the indices in the fast time. and can be represented as:

(1) $$p_{i}=s_{i}+n_{i}$$

(2) $$p_{j}=s_{j}+n_{j}$$

$s_{i}$ and $s_{j}$ are the target signals owing to the respiration; $n_{i}$ and $n_{j}$ are the zero mean additive white Gaussian noises. Calculating the cross-correlation between $p_{i}$ and $p_{j}$,

(3) $$\begin{array}{l} R_{p_{i}p_{j}}=E\left[p_{i}p_{j}\right]=E\left[\left(s_{i}+n_{i}\right)\left(s_{j}+n_{j}\right)\right]\\ =E\left[s_{i}s_{j}\right]+E\left[s_{i}n_{j}\right]+E\left[n_{i}s_{j}\right]+E\left[n_{i}n_{j}\right] \end{array}$$

As the signals are correlated and the noises are uncorrelated, we know that:

(4) $$E\left[s_{i}n_{j}\right]=E\left[n_{i}s_{j}\right]=E\left[n_{i}n_{j}\right]=0\;\left(i\neq j\right)$$

Finally, we can deduce $R_{p_{i}p_{j}}=\;\;E\left[s_{i}s_{j}\right]$, indicating that the degree by which $p_{i}$ and $p_{j}$ are correlated depends largely on the correlation between $s_{i}$ and $s_{j}$. If $s_{i}$ and $s_{j}$ arise from a single target, $R_{p_{i}p_{j}}$ is large; hence, it can be deduced that $R_{p_{i}p_{j}}$ is generally large in the correlation area for the same origination. To quantify the correlation between $p_{i}$ and $p_{j}$, the correlation coefficient is calculated as follows:

(5) $${R^{\prime }}_{p_{i}p_{j}}=\left|\frac{\sum_{t=1}^{N}\left(p_{i}\left(t\right)-\bar{p_{i}}\right)\times \left(p_{j}\left(t\right)-\bar{p_{j}}\right)}{\sqrt{\sum_{t=1}^{N}{\left(p_{i}\left(t\right)-\bar{p_{i}}\right)}^{2}}\sqrt{\sum_{t=1}^{N}{\left(p_{j}\left(t\right)-\bar{p_{j}}\right)}^{2}}}\right|$$

${R^{\prime }}_{p_{i}p_{j}}$ takes an absolute value because we focus on the strength of the correlation rather than on a positive or negative correlation. The correlation coefficients are computed between each row vector and the remaining row vectors in the matrix, P and the results are placed in a matrix, V, called the correlation coefficient matrix:

(6) $$V\left(i,j\right)={R^{\prime }}_{p_{i}p_{j}}$$

Thus, the correlation between every two of the received waveforms in P is determined quantitatively.

The matrix, V, has two characteristics:

(7) $$\begin{array}{cccc} V_{1\;1} & V_{1\;2} & \cdots & V_{1\;200}\\ V_{2\;1} & V_{2\;2} & \cdots & V_{2\;200}\\ \vdots & \vdots & \ddots & \vdots \\ V_{200\;1} & V_{200\;2} & \cdots & V_{200\;200} \end{array}$$

(1) It is a symmetric matrix, i.e.,
  (8) $$V_{i\;\;j}=V_{j\;i}\;\;\left(i\;\;j\in 1\cdots 200\right)$$
(2) The elements of the main diagonal in matrix, V, are all unity, i.e.,
  (9) $$V_{i\;j}=1\;\mathrm{if}\;i=j$$

**Figure 4:**
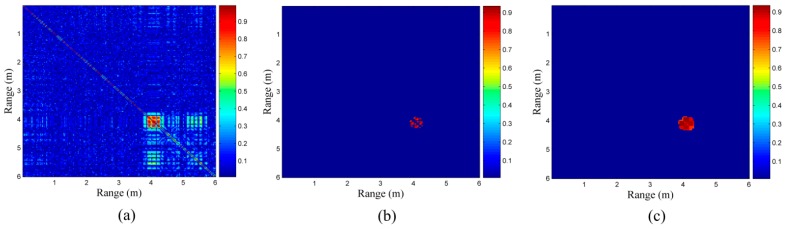
(**a**) correlation coefficient matrix of the data in Figure 3b, (**b**) region of interest reserved after processing, and (**c**) final correlation coefficient matrix.

Considering the single target in Figure 3a as an example, Figure 4a is its correlation coefficient matrix because the correlation coefficients in the correlation area are relatively large and the matrix is symmetrical. The area wherein the target is present mainly appears in the form of a red square along the diagonal line; as mentioned earlier, the signals are correlated and the noises are uncorrelated, and we know that the correlation coefficient in the region of interest where target is located is larger than those without a target nearby. If the area with the high correlation coefficients is recognized, the target identification problem can be resolved. In Figure 4a, we need to identify the red square along the diagonal line.

Two parameters are set to quantify the above requirement. The first is the width, W, of the region of interest because we will focus only on the given correlation area behind the target location, and the elements in the correlation coefficient matrix that are beyond the scope of the region of interest will be set to zero. Therefore, it is equivalent to slide a cross-section of a square with a side length, W, from one end to the other along the diagonal line, V; this is the cross-area that is intended to be reserved. Following this step, a new correlation coefficient matrix, J, is obtained as follows:

(10) $$\frac{W}{200}=\frac{T}{D}\Rightarrow W=\left\lfloor \frac{200\times T}{D}\right\rfloor$$

(11) $$J_{i\;\;j}=\left\{ \begin{array}{ll} V_{i\;\;j} & |i-j|\leq W\\ 0 & |i-j|>W \end{array}\right.$$

T is the approximate thoracic thickness of a normal adult, generally, T=0.24 m; D is the time window width. The second is the threshold that is employed for distinguishing a high correlation from a low correlation. The threshold is set mainly based on experience; in the actual experiment, as the elements of the main diagonal in the correlation coefficient matrix are all unity, representing the coefficients of autocorrelation, they should not be considered. The procedure is as follows:

(12) $$K_{i\;\;j}=\{\begin{array}{cc} J_{i\;\;j} & J_{i\;\;j}\geq 0.75\\ 0 & J_{i\;\;j}<0.75\cup i=j \end{array}$$

As shown in Figure 4b, the elements in the correlation coefficient matrix that are not less than 0.75 in the correlation area are reserved. Except for those along the diagonal line, what remains is what we are interested in.

Another problem that is to be addressed is that the region of interest is generally disconnected from the image recognition. A method that is similar to the dilatation algorithm is adopted during image processing to solve this problem. The all unity matrix, $A_{4\;\times \;4}$, is defined to traverse the matrix, K, as follows:

(13) $$F_{x\;\;y}=\frac{1}{G}\sum_{i=x}^{x+3}\sum_{j=y}^{y+3}K_{i\;\;j}\;\;(1\leq x\leq 197,1\leq y\leq 197)$$

G is the number of elements that are greater than or equal to 0.75 in the set, Z:

(14) $$Z=\left\{K_{i\;\;j}|x\leq i\leq x+3,y\leq j\leq y+3\right\}$$

In the final cut-through image shown in Figure 4c, the target located 4 m away from radar can be easily observed.

## 5. Experimental Results

This section presents the experimental results for the following three scenarios: no target, two targets, and three targets. A 24 cm thick brick wall is present in all these scenarios. We mainly focus on reducing the chances of false positives or false negatives that are common problems in target detection.

**Figure 5:**
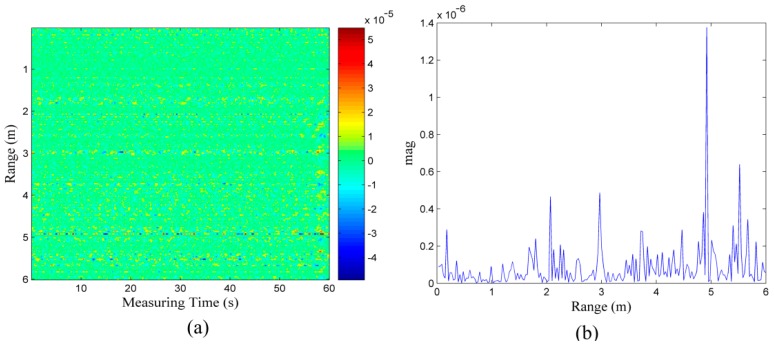

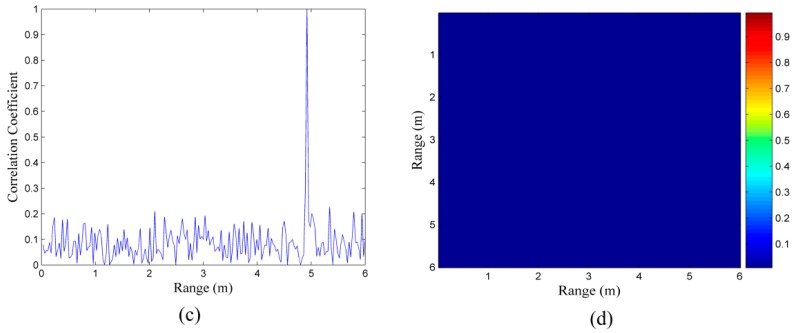
(**a**) data received from the scenario with no target; (**b**) energy of the data in (**a**); (**c**) correlation coefficients between the waveform at the peak energy location and the waveforms in the other locations; and (**d**) final correlation coefficient matrix.

In the first scenario, although there is no target in the detection area, as shown in Figure 5a, Figure 5b shows that the energy at the location situated 5 m from the radar is obviously greater than the other locations. The previous method of target identification based on the energy detection of the respiratory response is likely to result in a wrong judgment, i.e., the noise or clutter may be mistakenly considered a target. In Figure 5c that displays the correlation coefficients between the waveform at the 5 m location and the waveforms at other locations, we can find that the correlations are low, despite the fact that the strongest energy is present at the 5 m location. It appears to be random and independent as a correlation area does not exist at the 5 m location. Therefore, it is noise rather than breathing. Figure 5d shows the final result of our processing based on the cross-correlation, indicating that there is no target in the detection area because no red squares appear along the diagonal line of the correlation coefficient matrix; hence, false positives do not occur in our end result.

**Figure 6:**
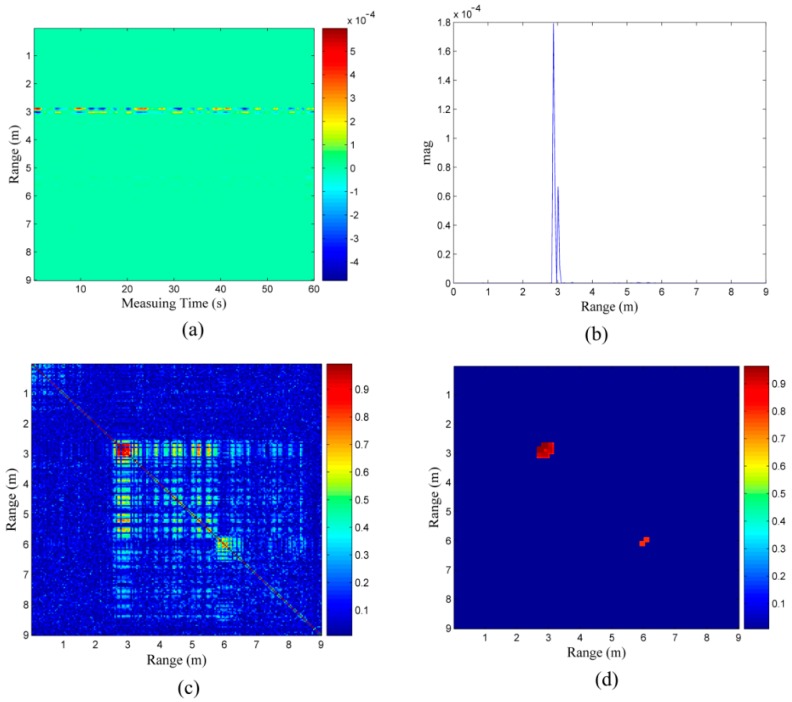
(**a**) data received from the scenario with the two target as shown in Figure 2a; (**b**) energy of the data in (**a**); (**c**) correla tion coefficient matrix of the data in (**a**); and (**d**) final correlation coefficient matrix.

As mentioned previously in Figure 2b, as the energy of target B is considerably subtle compared to target A, target B cannot be detected. This mainly because target B is located in the shadowing area caused by target A. In Figure 6a,b, we can find only one target, situated 3 m away from the radar, and the farther target at 6 m cannot be detected. The previous method based on the energy detection of the respiratory response produces an incorrect result with a false negative. In Figure 6c, the correlation coefficient matrix, we can find that the correlation coefficients of the region at 6 m are also large, although their correlation is smaller than those at the 3 m location. In the final result is shown in Figure 6d, there is an another obvious target at the 6 m location, besides the one at the 3 m location; therefore, the problem of the false negative is resolved.

**Figure 7:**
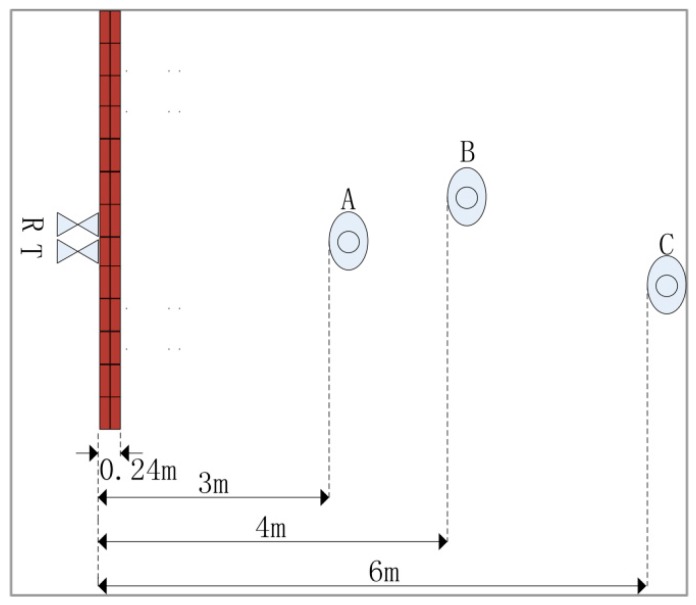
Scenario with three targets.

An experiment for detecting three targets was also performed. The distribution of targets A, B, and C is represented in Figure 7.

**Figure 8:**
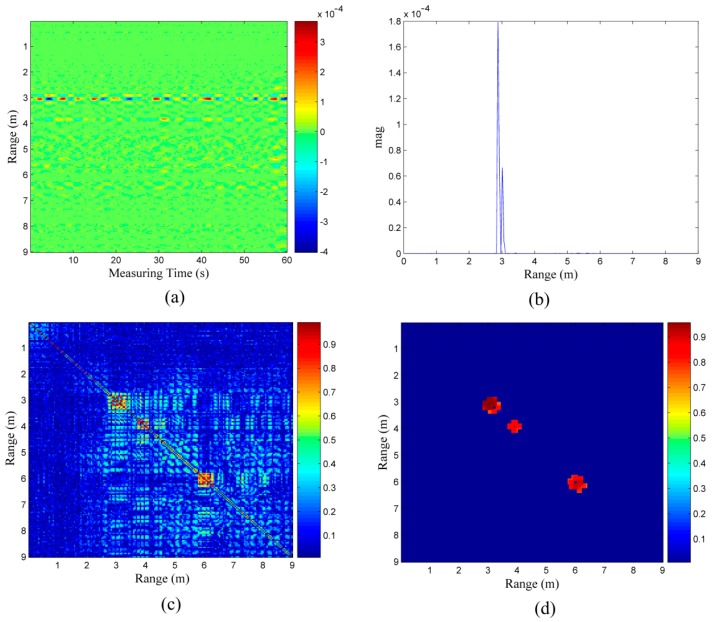
(**a**) data received from the scenario with the three target as shown in Figure 7; (**b**) energy of the data in (**a**); (**c**) correlation coefficient matrix of the data in (**a**); and (**d**) final correlation coefficient matrix.

There is only one obvious correlation area in Figure 8a; according to its energy characteristics in Figure 8b, the energy of target A at the 3 m location is considerably stronger than that of target B at the 4 m location and target C at the 6 m location. This is mainly because target A is facing the radar and is the closest. Though there is peak energy at the 4 m location also, it may be caused by target A owing to the trailing effect; whether it is a human target needs to be confirmed. In the correlation coefficient matrix shown in Figure 8c, we can roughly determine three targets located at 3 m, 4 m, and 6 m separately. In the final result is shown in Figure 8d, the locations of targets A, B, and C are immediately clear.

The above-mentioned experiments have been performed for three scenarios: no target, two targets, and three targets. The problem of the false positives and negatives encountered previously is well resolved based on the cross-correlation method. This is mainly because this method does not rely on the strength of the target energy. The farther targets can be identified after processing, even though their energies are considerably subtle for detection.

## 6. Conclusions

In this paper, a new target detection and identification method based on cross-correlation has been presented for solving the problem of the false negative, when a farther target is located in the shadowing area generated by a closer target. The previous detection method based on the energy detection of the respiratory response could not find the farther target in this scenario because the energy of the farther target is generally subtler by comparison. The characteristic of the correlation has been investigated, while determining the presence of the correlation area. Based on the characteristic found in the correlation coefficient matrix, a new algorithm was adopted for determining the final region of interest.

Four scenario experiments have been performed, with no target, a single target, two targets, and three targets. The single target scenario experiment was mainly used to extract the feature of the correlation coefficient matrix that enables us to address the issue of the target identification by searching for the high correlation region, i.e., the red square along the diagonal line of the correlation coefficient matrix. In the other three scenario experiments, based on the cross-correlation method, the problems of the false positives and false negatives could be solved effectively. The results indicate that the precision of the detection and identification of multiple stationary targets has clearly improved.

Further work will focus on searching the area of interest automatically, rather than depending on the threshold of 0.75 that was set based on experience.
